# SARS-CoV-2 viral load in nasopharyngeal swabs is not an independent predictor of unfavorable outcome

**DOI:** 10.1038/s41598-021-92400-y

**Published:** 2021-06-21

**Authors:** Sonsoles Salto-Alejandre, Judith Berastegui-Cabrera, Pedro Camacho-Martínez, Carmen Infante-Domínguez, Marta Carretero-Ledesma, Juan Carlos Crespo-Rivas, Eduardo Márquez, José Manuel Lomas, Claudio Bueno, Rosario Amaya, José Antonio Lepe, José Miguel Cisneros, Jerónimo Pachón, Elisa Cordero, Javier Sánchez-Céspedes, The Virgen del Rocío Hospital COVID-19 Working Team, José Miguel Cisneros, Manuela Aguilar-Guisado, Almudena Aguilera, Clara Aguilera, Teresa Aldabo-Pallas, Verónica Alfaro-Lara, Cristina Amodeo, Javier Ampuero, María Dolores Avilés, Maribel Asensio, Bosco Barón-Franco, Lydia Barrera-Pulido, Rafael Bellido-Alba, Máximo Bernabeu-Wittel, Candela Caballero-Eraso, Macarena Cabrera, Enrique Calderón, Jesús Carbajal-Guerrero, Manuela Cid-Cumplido, Yael Corcia-Palomo, Juan Delgado, Antonio Domínguez-Petit, Alejandro Deniz, Reginal Dusseck-Brutus, Ana Escoresca-Ortega, Fátima Espinosa, Nuria Espinosa, Michelle Espinoza, Carmen Ferrándiz-Millón, Marta Ferrer, Teresa Ferrer, Ignacio Gallego-Texeira, Rosa Gámez-Mancera, Emilio García, Horacio García-Delgado, Manuel García-Gutiérrez, María Luisa Gascón-Castillo, Aurora González-Estrada, Demetrio González, Carmen Gómez-González, Rocío González-León, Carmen Grande-Cabrerizo, Sonia Gutiérrez, Carlos Hernández-Quiles, Inmaculada Concepción Herrera-Melero, Marta Herrero-Romero, Luis Jara, Carlos Jiménez-Juan, Silvia Jiménez-Jorge, Mercedes Jiménez-Sánchez, Julia Lanseros-Tenllado, Carmina López, Isabel López, Álvaro López-Barrios, Luis F. López-Cortés, Rafael Luque-Márquez, Daniel Macías-García, Guillermo Martín-Gutiérrez, Luis Martín-Villén, José Molina, Aurora Morillo, María Dolores Navarro-Amuedo, Dolores Nieto-Martín, Francisco Ortega, María Paniagua-García, Amelia Peña-Rodríguez, Esther Pérez, Manuel Poyato, Julia Praena-Segovia, Rafaela Ríos, Cristina Roca-Oporto, Jesús F. Rodríguez, María Jesús Rodríguez-Hernández, Santiago Rodríguez-Suárez, Ángel Rodríguez-Villodres, Nieves Romero-Rodríguez, Ricardo Ruiz, Zaida Ruiz de Azua, Celia Salamanca, Sonia Sánchez, Víctor Manuel Sánchez-Montagut, César Sotomayor, Alejandro Suárez Benjumea, Javier Toral

**Affiliations:** 1 Unit of Infectious Diseases, Microbiology, and Preventive Medicine, Virgen del Rocío University Hospital, Seville, Spain; 2 Institute of Biomedicine of Seville (IBiS), Virgen del Rocío University Hospital/CSIC/University of Seville, Seville, Spain; 3 Medico-Surgical Unit of Respiratory Diseases, Virgen del Rocío University Hospital, Seville, Spain; 4 Unit of Emergencies, Virgen del Rocío University Hospital, Seville, Spain; 5 Intensive Care Unit, Virgen del Rocío University Hospital, Seville, Spain; 6 Department of Medicine, University of Seville, Seville, Spain; 7 Cardiology and Cardiovascular Surgery Unit, Virgen del Rocío University Hospital, Seville, Spain; 8 Rheumatology Unit, Virgen del Rocío University Hospital, Seville, Spain; 9 Internal Medicine Unit, Virgen del Rocío University Hospital, Seville, Spain; 10 Digestive and Hepatobiliary Unit, Virgen del Rocío University Hospital, Seville, Spain; 11 Neurology and Clinical Neurophysiology Unit, Virgen del Rocío University Hospital, Seville, Spain; 12 Endocrinology and Nutrition Unit, Virgen del Rocío University Hospital, Seville, Spain; 13 Urology and Nephrology Unit, Virgen del Rocío University Hospital, Seville, Spain; 14 Unit of Clinical Investigation and Clinical Trials, Virgen del Rocío University Hospital, Seville, Spain

**Keywords:** Risk factors, Viral infection

## Abstract

The aim was to assess the ability of nasopharyngeal SARS-CoV-2 viral load at first patient’s hospital evaluation to predict unfavorable outcomes. We conducted a prospective cohort study including 321 adult patients with confirmed COVID-19 through RT-PCR in nasopharyngeal swabs. Quantitative Synthetic SARS-CoV-2 RNA cycle threshold values were used to calculate the viral load in log_10_ copies/mL. Disease severity at the end of follow up was categorized into mild, moderate, and severe. Primary endpoint was a composite of intensive care unit (ICU) admission and/or death (n = 85, 26.4%). Univariable and multivariable logistic regression analyses were performed. Nasopharyngeal SARS-CoV-2 viral load over the second quartile (≥ 7.35 log_10_ copies/mL, *p* = 0.003) and second tertile (≥ 8.27 log_10_ copies/mL, *p* = 0.01) were associated to unfavorable outcome in the unadjusted logistic regression analysis. However, in the final multivariable analysis, viral load was not independently associated with an unfavorable outcome. Five predictors were independently associated with increased odds of ICU admission and/or death: age ≥ 70 years, SpO_2_, neutrophils > 7.5 × 10^3^/µL, lactate dehydrogenase ≥ 300 U/L, and C-reactive protein ≥ 100 mg/L. In summary, nasopharyngeal SARS-CoV-2 viral load on admission is generally high in patients with COVID-19, regardless of illness severity, but it cannot be used as an independent predictor of unfavorable clinical outcome.

## Introduction

The novel severe acute respiratory syndrome coronavirus 2 (SARS-CoV-2), causative agent of coronavirus disease 2019 (COVID-19), has spread worldwide, becoming a pandemic of historic dimensions^1^. The clinical spectrum of COVID-19 ranges from asymptomatic disease to pneumonia, life-threatening complications, and, ultimately, death^2,3^. Despite most infected individuals develop solely a mild illness, the mortality rate for severe cases is as high as that caused by other etiologies of severe community-acquired pneumonia^4^.

For coping with the best clinical attention to COVID-19 patients it is crucial to perform prognosis estimations at the first clinical evaluation, offering personalized attention based on early and easily detectable predictors that support decision making, guide level of care, and optimize the allocation of health resources. Different studies have already addressed this issue, identifying clinical signs and several biomarkers as predictors of unfavorable outcome^5–7^.

In this regard, different studies have addressed the possible association between the viral load in nasopharyngeal (NP) swabs and the clinical outcomes. Some studies have reported that a high number of virus copies in NP swabs, mainly defined as a cycle threshold (Ct) < 25 or < 22 in the real-time polymerase chain reaction (RT-PCR), was an independent risk factor for intubation and/or death^8–11^. However, other studies have not found independent association between low Ct values and critical care admission or death^12,13^. In short, the real impact of initial SARS-CoV-2 viral load in NP swabs on COVID-19 patients’ outcomes is not been fully elucidated, and this issue remains controversial^14^.

In the present prospective study on adult COVID-19 patients, stratified into mild disease (attended as outpatients) and hospital admitted with moderate or severe disease, we analyzed if the viral load of SARS-CoV-2 in NP swabs was associated with the disease severity, and the ability of NP SARS-CoV-2 viral load at the first hospital evaluation to predict unfavorable outcomes.

## Results

### Demographics, clinical characteristics, and outcome

The cohort included 321 adult patients, with the first evaluation at the Emergency room. Fifty-six (17.4%) patients had a mild disease and were discharged after the first evaluation, and subsequently attended as outpatients until the end of follow-up; 180 (56.1%) had a moderate course, being hospitalized in general wards, and with full recovery and hospital discharged; and 85 (26.5%) patients were categorized as severe COVID-19 because of required admission to the ICU (32 patients [10.0%]), in-hospital death (40 [12.5%]), or both (13 [4.0%]).

Demographics, symptoms, and signs of the total cohort and the three categories of disease severity are shown in Table 1. In the total cohort, males accounted for 169 (52.6%), median age was 63 (IQR 52–77) years, and 36.8% were ≥ 70 years old. The most common symptoms were fever (73.8%), cough (67.3%), and dyspnea (45.8%). Two hundred twenty-four (69.8%) patients had chest X-ray infiltrates at first hospital evaluation: 16.1% within the mild group, 77.8% in the moderate one, and 88.2% in the severe group (*p* < 0.001). During the follow-up, 100% of the patients in the moderate and severe groups showed pulmonary infiltrates in the evolutive chest X-ray after hospital admission. Between-group differences regarding baseline laboratory values were also identified and are detailed in Table 2.

**Table 1:** Demographics, comorbidities, and clinical data of 321 patients with COVID-19 stratified according to disease severity.

	Total cohort (n = 321)	Mild disease (n = 56)	Moderate disease (n = 180)	Severe disease (n = 85)	*p* value^c^
Age in years, median (IQR)	63 (52–77)	48 (40–60)	62 (52–75)	75 (63–84)	< 0.001
Age ≥ 70 (%)	118 (36.8)	8 (14.3)	58 (32.2)	52 (61.2)	< 0.001
Male sex (%)	169 (52.6)	29 (51.8)	90 (50.0)	50 (58.8)	0.40
Chronic underlying diseases (%)					
Arterial hypertension	150 (46.7)	16 (28.6)	80 (44.4)	54 (63.5)	< 0.001
Diabetes mellitus	57 (17.8)	6 (10.7)	35 (19.4)	16 (18.8)	0.31
Chronic lung disease^a^	38 (11.8)	3 (5.4)	26 (14.4)	9 (10.6)	0.17
Cardiovascular disease	64 (19.9)	5 (8.9)	32 (17.8)	27 (31.8)	0.002
Chronic kidney disease	22 (6.9)	2 (3.6)	13 (7.2)	7 (8.2)	0.54
Chronic liver disease	10 (3.1)	1 (1.8)	6 (3.3)	3 (3.5)	0.82
Cancer^b^	25 (7.8)	5 (8.9)	9 (5.0)	11 (12.9)	0.08
Symptoms (%)					
Fever	237 (73.8)	38 (67.9)	142 (78.9)	57 (67.1)	0.07
Rhinorrhea	19 (5.9)	6 (10.7)	10 (5.6)	3 (3.5)	0.20
Odynophagia	22 (6.9)	6 (10.7)	11 (6.1)	5 (5.9)	0.45
Myalgias	70 (21.8)	13 (23.2)	37 (20.6)	20 (23.5)	0.83
Headache	57 (17.8)	11 (19.6)	30 (16.7)	16 (18.8)	0.84
Cough	216 (67.3)	40 (71.4)	128 (71.1)	48 (56.5)	0.04
Expectoration	33 (10.3)	7 (12.5)	16 (8.9)	10 (11.8)	0.64
Pleuritic chest pain	14 (4.4)	3 (5.4)	9 (5.0)	2 (2.4)	0.57
Dyspnea	147 (45.8)	19 (33.9)	75 (41.7)	53 (62.4)	0.001
Diarrhea	52 (16.2)	12 (21.4)	33 (18.3)	7 (8.2)	0.06
Vomiting	20 (6.2)	1 (1.8)	16 (8.9)	3 (3.5)	0.08
Impaired consciousness	12 (3.7)	1 (1.8)	6 (3.3)	5 (5.9)	0.42
Days from symptom onset to diagnosis, median (IQR)	7 (3–10)	7 (5–12)	6 (3–10)	6 (2–10)	0.35
Infiltrate on chest X-ray (%)	224 (69.8)	9 (16.1)	140 (77.8)	75 (88.2)	< 0.001
Signs (categorized, %)					
Temperature > 37.5 °C	82 (26.9)	5 (11.1)	48 (27.0)	29 (35.4)	0.01
SBP < 90 mmHg	7 (2.5)	1 (2.4)	4 (2.4)	2 (2.8)	0.99
DBP < 60 mmHg	24 (8.6)	1 (2.4)	9 (5.5)	14 (19.4)	0.001
Hart rate > 100 bpm	70 (21.8)	5 (8.9)	41 (22.8)	24 (28.2)	0.02
Respiratory rate > 20 bpm	17 (20.0)	0 (0)	5 (10.6)	12 (35.3)	0.01
SpO_2_ < 95%	127 (39.6)	4 (7.1)	52 (28.9)	71 (83.5)	< 0.001

^a^Chronic obstructive pulmonary disease, obstructive sleep apnea, or asthma.

^b^Active solid or hematologic malignant neoplasms.

^c^Across all three groups.

**Table 2:** Laboratory values and nasopharyngeal SARS-CoV-2 viral load of 321 patients with COVID-19 stratified according to disease severity.

	Total cohort (n = 321)	Mild disease (n = 56)	Moderate disease (n = 180)	Severe disease (n = 85)	*p* value^b^
**Blood counts, median (IQR)**					
WBC × 10^3^/µL	6.5 (4.7–9.0)	5.0 (4.0–7.0)	6.5 (4.8–8.7)	8.1 (5.3–11.7)	< 0.001
Neutrophils × 10^3^/µL	4.7 (3.2–7.1)	3.4 (2.4–4.7)	4.6 (3.2–6.7)	6.9 (4.0–9.9)	0.64
Lymphocytes × 10^3^/µL	1.1 (.8–1.6)	1.3 (1.1–1.6)	1.1 (.8–1.6)	.9 (.6–1.4)	< 0.001
Platelets × 10^3^/µL	198 (163–257)	202 (164–243)	197 (165–253)	200 (161–268)	0.70
**Blood counts (categorized, %)**					
WBC > 11 × 10^3^/µL	42 (13.1)	1 (1.8)	16 (8.9)	25 (29.4)	< 0.001
Neutrophils > 7.5 × 10^3^/µL	65 (20.2)	0 (0)	31 (17.2)	34 (40.0)	< 0.001
Lymphocytes < 1 × 10^3^/µL	125 (38.9)	7 (12.5)	68 (37.8)	50 (58.8)	< 0.001
Platelets < 130 × 10^3^/µL	26 (8.1)	5 (8.9)	11 (6.1)	10 (11.8)	0.28
**Biochemistry and inflammatory biomarkers, median (IQR)**					
Creatinine mg/dL	.9 (.7–1.2)	.8 (.7–1.0)	.9 (.7–1.1)	1.1 (.8–1.6)	0.42
AST U/L	29 (22–49)	24 (18–32)	27 (21–46)	39 (28–64)	0.01
LDH U/L	309 (231–415)	222 (185–280)	293 (229–376)	400 (319–502)	< 0.001
CRP mg/L^﻿a^	57.0 (20.8–136.6)	16.0 (5.8–33.9)	53.0 (20.0–113.8)	142.5 (67.2–252.0)	< 0.001
D-dimer ng/mL^a^	770 (463–1608)	515 (345–755)	730 (428–1578)	1145 (708–2453)	0.07
**Biochemistry and inflammatory biomarkers (categorized, %)**					
Creatinine > 1.3 mg/dL	60 (18.7)	4 (7.1)	28 (15.6)	28 (32.9)	< 0.001
AST > 30 U/L	124 (38.6)	11 (19.6)	62 (34.4)	51 (60.0)	< 0.001
LDH ≥ 300 U/L	145 (45.2)	4 (7.1)	79 (43.9)	62 (72.9)	< 0.001
CRP ≥ 100 mg/L﻿^a^	102 (31.8)	1 (1.8)	52 (28.9)	49 (57.6)	< 0.001
D-dimer ≥ 600 ng/mL^﻿﻿a^﻿	171 (53.3)	13 (23.2)	97 (53.9)	61 (71.8)	< 0.001
**Nasopharyngeal viral load (log**_**10**_** copies/mL, median [IQR])**					
Viral load (VL)	7.35 (5.85–8.80)	6.44 (4.70–8.32)	7.10 (5.92–8.66)	8.18 (6.31–8.90)	0.88
VL ≥ 6.33 (1st tertile, %)	215 (67.0)	29 (51.8)	122 (67.8)	64 (75.3)	0.01
VL ≥ 7.35 (50th percentile, %)	163 (50.8)	24 (42.9)	84 (46.7)	55 (64.7)	0.01
VL ≥ 8.27 (2nd tertile, %)	107 (33.3)	16 (28.6)	52 (28.9)	39 (45.9)	0.02

^a^Values were available in 293 and 276 patients for CRP and D-dimer, respectively.

^b^Across all three groups.

Seventy-eight (24.3%) patients required respiratory support with high flow therapy or non-invasive mechanical ventilation, which was more frequent in patients with severe than with moderate disease (55 [64.7%] vs. 23 [12.8%], respectively, being *p* = 0.001). Twenty-eight (32.9%) patients, all admitted to ICU, required invasive mechanical ventilation.

Median NP viral load at first hospital evaluation was not different among the mild, moderate, or severe groups according to their clinical outcomes (Table 2). However, we found higher frequencies of NP viral load above the first tertile, the 50th percentile, and the second tertile in the severe group when comparing the three groups (*p* = 0.01).

We also analyzed the possible differences among demographics, chronic underlying diseases, and the days from symptoms onset to diagnosis according to the SARS-CoV-2 viral load (Table 3). Although the median days from symptoms onset to diagnosis was lower in the group with higher SARS-CoV-2 viral load (2nd vs. 1st tertile) this difference was not significant. Additionally, we performed a linear regression analysis which did not show association between both variables (*p* = 0.389). The only significant differences (*p* < 0.05) were found for the frequency of cardiovascular diseases and age ≥ 70 years. Finally, we made a linear regression analysis between the SARS-CoV-2 viral load and age, finding a significant correlation between both variables (*p* < 0.001) though not clinically relevant (adjusted *R*^*2*^ 0.036).

**Table 3:** Demographics, comorbidities, and days from symptoms onset to diagnosis of 321 patients with COVID-19 stratified according to nasopharyngeal viral load (VL, log_10_ copies/mL).

	VL ≤ 6.33 (1st tertile) (n = 107)	VL 6.34–8.26 (1st to 2nd tertile) (n = 107)	VL ≥ 8.27 (2nd tertile) (n = 107)	*p* value
Age ≥ 70 years	27 (25.2)	40 (37.4)	51 (47.7)	0.003
Male sex	57 (53.3)	64 (59.8)	48 (44.9)	0.09
Arterial hypertension	40 (37.4)	57 (53.3)	53 (49.5)	0.05
Diabetes mellitus	22 (20.6)	14 (13.1)	21 (19.6)	0.30
Chronic lung disease^a^	12 (11.2)	11 (10.3)	15 (14.0)	0.68
Cardiovascular disease	19 (17.8)	15 (14.0)	30 (28.0)	0.03
Chronic kidney disease	5 (4.7)	7 (6.5)	10 (9.3)	0.40
Chronic liver disease	4 (3.7)	4 (3.7)	2 (1.9)	0.66
Cancer^b^	9 (8.4)	8 (7.5)	8 (7.5)	0.96
Days from symptom onset to diagnosis, median (IQR)	7 (5–12)	7 (4–10)	4 (1–7)	0.27^c^

Data are presented as n (%) unless otherwise indicated.

^a^Chronic obstructive pulmonary disease, obstructive sleep apnea, or asthma.

^b^Active solid or hematologic malignant neoplasms.

^c^Viral load ≤ 6.33 (1st tertile) vs. viral load ≥ 8.27 (2nd tertile).

### Predictors of unfavorable outcome

Twenty-three categorical variables at first hospital evaluation were identified as baseline risk factors for unfavorable outcome (admission to ICU or death) in the unadjusted logistic regression analysis: advanced age, arterial hypertension, cardiovascular disease, cancer, dyspnea, higher temperature and respiratory rate, lower diastolic blood pressure and capillary oxygen saturation, leukocytes > 11 × 10^3^/µL, neutrophils > 7.5 × 10^3^/µL, lymphocytes < 1 × 10^3^/µL, and higher levels of creatinine, aspartate aminotransferase, lactate dehydrogenase (LDH), C-reactive protein (CRP), and D-dimer, among others (Table 4). Regarding the NP viral load, values over the second quartile and second tertile were also associated with unfavorable outcome in the unadjusted logistic regression analysis (Table 4).

**Table 4:** Baseline risk factors for unfavorable outcome (intensive care unit admission and/or death): Univariable logistic regression analysis.

	Crude odds ratio (95% CI)	*p* value
Age ≥ 70 years	4.06 (2.41–6.83)	< 0.001
Arterial hypertension	2.54 (1.52–4.24)	< 0.001
Cardiovascular disease	2.50 (1.41–4.45)	0.002
Cancer	2.36 (1.03–5.42)	0.043
Cough	.53 (.31-.88)	0.01
Dyspnea	2.50 (1.50–4.17)	< 0.001
Diarrhea	.38 (.17-.88)	0.02
Infiltrate on chest X-ray	4.38 (2.15–8.92)	< 0.001
Temperature > 37.5 °C	1.76 (1.02–3.04)	0.04
DBP < 60 mmHg	4.73 (2.00–11.21)	< 0.001
Respiratory rate > 20 bpm	5.02 (1.57–16.01)	0.006
SpO_2_ < 95%	16.30 (8.54–31.13)	< 0.001
WBC > 11 × 10^3^/µL	5.37 (2.72–10.59)	< 0.001
Neutrophils > 7.5 × 10^3^/µL	4.41 (2.48–7.84)	< 0.001
Lymphocytes < 1 × 10^3^/µL	3.07 (1.84–5.12)	< 0.001
Creatinine > 1.3 mg/dL	3.13 (1.74–5.63)	< 0.001
AST > 30 U/L	3.35 (2.00–5.60)	< 0.001
LDH ≥ 300 U/L	4.97 (2.87–8.60)	< 0.001
CRP ≥ 100 mg/L	4.70 (2.77–7.97)	< 0.001
D-dimer ≥ 600 ng/mL	2.91 (1.70–4.98)	< 0.001
Viral load ≥ 7.35 log_10_ copies/mL (50th percentile)	2.17 (1.30–3.63)	0.003
Viral load ≥ 8.27 log_10_ copies/mL (2nd tertile)	2.10 (1.26–3.49)	0.01

**Figure 1:**
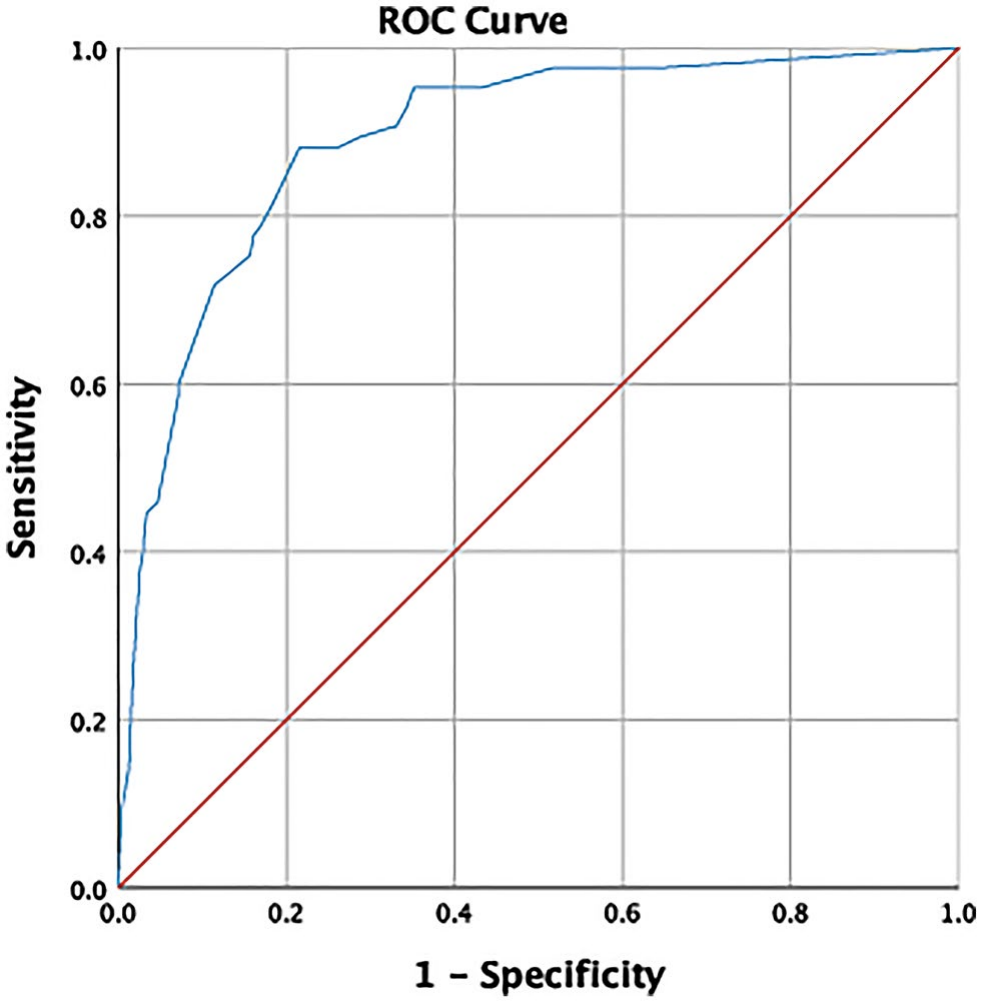
Discrimination power of the final multivariable model: ROC Curve plot. Discrimination power of the model (including Age ≥ 70 years, SpO_2_ < 95%, neutrophils > 7.5 × 10^3^/µL, LDH ≥ 300 U/L, and CRP ≥ 100 mg/L) expressed by an area under the ROC Curve of 0.89 (95% CI 0.85–0.93), standard error of 0.02 (under the non-parametric assumption), and *p* < 0.001 (being the null hypothesis a true area = 0.50).

In the final multivariable analysis, despite the previous link between a higher viral load and the occurrence of unfavorable outcome, the number of virus copies in the NP swabs was not independently associated with an unfavorable clinical result (Table 5). Five of the previous predictors were independently associated with increased odds of ICU admission and/or death: age ≥ 70 years (odds ratio [OR] 3.58, *p* < 0.001), SpO_2_ < 95% (OR 11.07, *p* < 0.001), neutrophils > 7.5 × 10^3^/µL (OR 3.67, *p* = 0.001), LDH ≥ 300 U/L (OR 2.11, *p* = 0.04), and CRP ≥ 100 mg/L (OR 2.61, *p* = 0.01). Information on the overall apparent performance of the model is presented in Table 5 and Fig. 1.

**Table 5:** Independent predictors of unfavorable outcome (ICU admission and/or death): Multivariable logistic regression model.

	Adjusted odds ratio (95% CI)	*p* value
Age ≥ 70 years	3.58 (1.83–6.99)	< 0.001
SpO_2_ < 95%	11.07 (5.34–22.97)	< 0.001
Neutrophils > 7.5 × 10^3^/µL	3.67 (1.74–7.74)	0.001
LDH ≥ 300 U/L	2.11 (1.04–4.31)	0.04
CRP ≥ 100 mg/L	2.61 (1.32–5.14)	0.01
Viral load ≥ 7.35 log_10_ copies/mL (50th percentile)	1.49 (.75–2.96)	0.25
Viral load ≥ 8.27 log_10_ copies/mL (2nd tertile)	1.84 (.92–3.68)	0.09

The final multivariable model was composed of five variables (therefore 17 events per variable) demonstrated as independent predictors of unfavorable outcome: Age ≥ 70 years, SpO_2_ < 95%, neutrophils > 7.5 × 10^3^/µL, LDH ≥ 300 U/L, and CRP ≥ 100 mg/L). Such model reported a Beta Coefficient of -4.08 (standard error = 0.46), a Wald statistic of 78.72 (degrees of freedom = 1), and an overall apparent performance of 84.2% (sensitivity = 70.6%, specificity = 89.4%, PPV = 70.3%, NPV = 89.1%). The variables included were explanatory and contributed to giving the model an ability to explain roughly 52.1% of the variation of the outcome (Nagelkerke *R*^2^ value = 0.521). A higher nasopharyngeal viral load (above the second quartile or the second tertile) was not independently linked to an increased risk of ICU admission or death.

## Discussion

The main finding of the present study is that patients with SARS-CoV-2 infection, regardless of their illness severity, generally have a high rate of viral replication in the upper respiratory airways. Consequently, this parameter cannot be used as a predictor of COVID-19 unfavorable outcome, defined as admission to ICU and/or death. Moreover, this prospective cohort confirms that the independent risk factors for ICU admission or death are those previously identified by Salto-Alejandre S. *et al*^7^. Thus, at first hospital evaluation, advanced age, hypoxemia, neutrophilia, and increased levels of LDH and CRP have high sensitivity and specificity to accurately discriminate patients that would potentially develop a critical disease from those with a favorable course.

The evidence to date reveals that the relationship between SARS-CoV-2 viral load and the pathogenicity and virulence of this microorganism is not fully understood. Furthermore, as there are many methods to perform the molecular detection of SARS-CoV-2 genome, the interpretation and comparison of results in literature is highly controversial. As an example, droplet digital PCR (ddPCR) has shown slightly higher sensitivity than standard RT-PCR^15^.

Several previous studies have demonstrated that a high value of SARS-CoV-2 viral load in the upper respiratory tract (URT), defined as a Ct < 25 or < 22 in the RT-PCR, is an independent risk factor for respiratory failure^8^, intubation, or death^9^, using multivariate logistic regression and time-based analyses^10^. Pujadas et al*.*, using a Cox proportional hazards model, also showed an independent association between viral load in the URT and mortality^11^.

Such results have often led to the thought that viral load could be used along with other features to decide upon the need for hospital admission, and even that a stratification for baseline NP viral load would benefit the design of clinical trials. Nevertheless, other studies show contradictory results. Maltezou et al*.*, using a Ct < 25 to define high URT viral load, reported an association between higher viral load and the development of COVID-19 disease, while no association was found with ICU admission, mechanical ventilation or death^12^. Amodio et al*.* demonstrated that the median PCR Ct was significantly lower in patients who died or needed critical care than in those who were hospitalized and discharged alive, or exclusively attended at home, but after adjusting for age and sex, there was not and independent association with critical care need or death^13^.

Similarly, in our study, despite the patients with higher viral load (above the first tertile, the 50th percentile, and the second tertile) often belonged to the severe disease group, the adjusted multivariable model did not find an association between the copies per mL and the need for critical care or mortality. Argyropoulos et al., on the other hand, showed that viral load was inversely correlated with disease severity, being higher in patients with mild COVID-19^16^. The reason for this conflictive result was, however, that NP sampling in patients with severe or critical symptoms was obtained at a later time point in the disease course. Lastly, Lee et al. found that viral load quantification was similar among symptomatic and asymptomatic patients^17^, and our results support this conclusion. Certainly, most patients in the present cohort who suffered an unfavorable outcome had a high SARS-CoV-2 viral load quantification (above the 50th percentile) at hospital admission, but half of the patients with mild COVID-19 also exceeded said limit. This corroborates that the number of virus copies is not strongly related to COVID-19 prognosis.

To further test our hypothesis, we stratified the patients according to disease severity at the end of follow up, and having confirmed that the medium time from symptoms onset to diagnosis was similar among groups, we performed multiple comparations for each viral load cut-off value. Mild patients could only be distinguished through the first tertile, above which a small increment of moderate and severe cases was found. For higher viral load cut-off points, the probability of belonging to the mild or moderate group was similar. The percentage of patients having NP viral load over the 50th percentile and second tertile was significantly higher for those with severe COVID-19, and the univariable analysis showed that a viral load quantification over the mentioned levels could be a risk factor for ICU admission or death. However, through the multivariable model, we concluded that a high viral load could not be used as an independent predictor of such outcomes.

Our study highlights several substantial issues. First, there is not a clear viral load cut-off point capable of discriminating between the various levels of COVID-19 severity, as the ROC Curve analysis demonstrated. Secondly and contrary to expectations, a higher number of SARS-CoV-2 copies in NP swabs at first patient’s evaluation is not predictive of whether ICU admission or death might occur. Nevertheless, according to the Spanish nationwide seroepidemiological study, this finding should not be surprising: a third of the population with positive PCR was asymptomatic, and 20% of the seropositive symptomatic participants did not have previous SARS-CoV-2 genome detection^18^. Finally, through the external cohort validation of hypoxemia, neutrophilia, and increased levels of LDH and CRP as independent predictors of unfavorable outcome^7^, we contribute to the identification of higher-risk patients with COVID-19 in whom suitable and prompt management is vital.

The main strength of the present study is that a wide spectrum of COVID-19 severity, from mild symptomatic to critically ill patients, is represented in the analyzed cohort, allowing novel conclusions to be drawn about the efficacy and predictive reliability of previously studied clinical factors. The study has also some limitations. The viral load quantification in the URT samples, through NP swabs, was only performed at a single time point, and we have not data on the dynamics according to the clinical outcomes. Additional synchronous and longitudinal sampling from other sources, such as blood or stools, would have been important comparators. Regarding the quantification of SARS-CoV-2 viral load, further studies are required to refine the use of the standard and novel techniques, which is especially important due to variabilities in specimen collection, the lack of systematic quantification assays, and inconsistencies in protocols between different laboratories. Also, the lack of association of NP viral load with unfavorable outcome, should be confirmed when COVID-19 be caused by the new SARS-CoV-2 variants.

In summary, we found that higher values of SARS-CoV-2 viral load in NP samples at first hospital evaluation are more frequent in patients with unfavorable in-hospital outcome, but that a high viral load is not an independent risk factor for ICU admission or death among adult patients with COVID-19.

## Methods

### Design, patients, and data collection

We conducted a prospective observational cohort study in Virgen del Rocío University Hospital, a Spanish care-teaching center with 1177 beds (including 72 adult ICU beds). The study protocol was approved by the Ethics Committee of Virgen Macarena and Virgen del Rocío University Hospitals (C.I. 0771-N-20), and complied the Declaration of Helsinki. An informed consent was established as a mandatory requirement for all patients. Consecutive patients with confirmed COVID-19 by RT-PCR assay for SARS-CoV-2 in NP samples were enrolled, from February 29th to May 1st, 2020. Baseline was the date of first hospital evaluation. Follow-up censoring date was May 29th, 2020, for a minimum observation period in each patient of 28 days.

The clinical data source was the electronic medical record system. Variables registered included demographics, comorbidities, symptoms and signs at admission, baseline laboratory tests and chest X-ray findings, complications during hospitalization, and clinical outcome.

### SARS-CoV-2 infection diagnosis and viral load

SARS-CoV-2 total RNA was extracted from NP swabs using EZ1 Virus Mini Kit v2.0 (Qiagen Inc., Valencia, CA, USA) following manufacturer’s instruction. SARS-CoV-2 genomic RNA was amplified by LightCycler 96 Instrument (Roche, Germany) using CDC 2019-Novel Coronavirus (2019-nCoV) Real-Time RT-PCR Diagnostic Panel and the GoTaq® Probe 1-Step RT-qPCR System (Wisconsin, USA) following the CDC’s instructions. The Quantitative Synthetic SARS-CoV-2 RNA: ORF, E, N kit (ATCC, VA, USA) for each NP sample was run and Ct values were interpolated into the curve obtained to calculate the viral load in log_10_ copies/mL. The lower and upper limits of quantification for our RT-PCR were 3.9 and 8.9 log_10_ copies/mL, respectively.

### Statistical analysis

Primary endpoint was the occurrence of unfavorable outcome at the end of follow-up, defined as a composite of ICU admission and/or death. For analyzing the ability of NP SARS-CoV-2 viral load at first patient’s evaluation to predict an unfavorable outcome, the severity of COVID-19 at the end of the 28 days follow-up was categorized into (1) mild, patients exclusively attended as outpatients after the first hospital evaluation; (2) moderate, hospitalized with full recovery and discharged; and (3) severe, hospitalized and admitted to the ICU or dead.

A descriptive analysis of all data obtained was performed. Categorical variables were presented as n (%) and continuous as median (interquartile range [IQR]). We used the *χ*^2^-test, Fisher’s Exact Test, One-Way Analysis of Variance (ANOVA), Kruskal Wallis Test, Student’s *t*-test, or Welch’s *t*-test to compare between-group differences, and linear regression analysis to assess association between variables, as appropriate.

To examine the factors associated with unfavorable outcome, a univariable logistic regression analysis was performed. Additionally, bivariate correlations were thoroughly explored to account for potential confusion and interaction effects. To increase the applicability of our results within the scope of clinical practice, continuous variables were dichotomized based on normal ranges and cut-off values previously identified as predictors of unfavorable outcome^7^.

Regarding SARS-CoV-2 viral load, for which there is no prior clinical consensus for categorization, we tried to determine the optimal cut-off value through a ROC curve plot (figure not shown). However, all the points in the curve approached the diagonal segment, with an area underneath of 0.57 (close to the null value) that called into question the usefulness of this parameter. Finally, we decided to analyze viral load based on three prespecified cut-off points: the first tertile, the second quartile or 50th percentile, and the second tertile.

For identifying which of the predictors obtained from the univariable analysis were to be considered independent, a multivariable logistic regression model was built using three criteria to achieve the highest accuracy: relevance to clinical situation, statistical significance (*p* < 0.10), and adequate number of events to allow meaningful analysis. An automated backward stepwise selection was used for exclusion of variables, utilizing a probability threshold of 5%. The model was first assessed for sensitivity, specificity, positive and negative predictive values, and overall apparent performance. Secondly, the fraction of variance explained by said model was estimated through the Nagelkerke *R*^2^ value. The internal validity was finally evaluated using the area under the ROC curve, where ≥ 0.70 (being the null hypothesis a true area of 0.50) is considered as evidence of good discrimination ability.

### Ethics approval

The study protocol was approved by the Ethics Committee of Virgen Macarena and Virgen del Rocío University Hospitals (C.I. 0771-N-20) and complied the Declaration of Helsinki.

## Supplementary Information

Supplementary Information.

## Data Availability

All data generated or analyzed during this study are included in this published article (and its Supplementary Information files).
